# Evaluating and Enhancing Face Anti-Spoofing Algorithms for Light Makeup: A General Detection Approach

**DOI:** 10.3390/s24248075

**Published:** 2024-12-18

**Authors:** Zhimao Lai, Yang Guo, Yongjian Hu, Wenkang Su, Renhai Feng

**Affiliations:** 1 School of Immigration Administration (Guangzhou), China People’s Police University, Guangzhou 510663, China; laizhimao@cppu.edu.cn; 2 School of Automation, Guangdong University and Technology, Guangzhou 510006, China; 3 School of Electronic and Information Engineering, South China University of Technology, Guangzhou 510641, China; 202121013152@mail.scut.edu.cn; 4 School of Computer Science and Cyber Engineering, Guangzhou University, Guangzhou 510006, China; swk1004@gzhu.edu.cn; 5 School of Electrical and Information Engineering, Tianjin University, Tianjin 300072, China; fengrenhai@tju.edu.cn

**Keywords:** deep neural network, face anti-spoofing, makeup transfer, metric learning, general detection

## Abstract

Makeup modifies facial textures and colors, impacting the precision of face anti-spoofing systems. Many individuals opt for light makeup in their daily lives, which generally does not hinder face identity recognition. However, current research in face anti-spoofing often neglects the influence of light makeup on facial feature recognition, notably the absence of publicly accessible datasets featuring light makeup faces. If these instances are incorrectly flagged as fraudulent by face anti-spoofing systems, it could lead to user inconvenience. In response, we develop a face anti-spoofing database that includes light makeup faces and establishes a criterion for determining light makeup to select appropriate data. Building on this foundation, we assess multiple established face anti-spoofing algorithms using the newly created database. Our findings reveal that the majority of these algorithms experience a decrease in performance when faced with light makeup faces. Consequently, this paper introduces a general face anti-spoofing algorithm specifically designed for light makeup faces, which includes a makeup augmentation module, a batch channel normalization module, a backbone network updated via the Exponential Moving Average (EMA) method, an asymmetric virtual triplet loss module, and a nearest neighbor supervised contrastive module. The experimental outcomes confirm that the proposed algorithm exhibits superior detection capabilities when handling light makeup faces.

## 1. Introduction

Face recognition technology has become widely utilized across various societal domains; however, it remains susceptible to a range of adversarial attacks. Consequently, research on face anti-spoofing technology is crucial for ensuring the secure and practical deployment of face recognition systems in real-world scenarios. Early face anti-spoofing methods primarily relied on traditional hand-crafted features, necessitating the manual design of operators to extract relevant features. This approach was labor-intensive, sensitive to environmental factors, and exhibited poor generalization performance. With the rapid advancement of deep learning, Yang et al. [1] pioneered the application of Convolutional Neural Networks (CNNs) in face anti-spoofing. Since then, an increasing number of researchers have focused on developing deep learning-based face anti-spoofing algorithms [2,3,4,5]. These methods address the limitations of traditional hand-crafted feature approaches by eliminating the need for manual operator design and can automatically learn facial features given a target function, thereby demonstrating superior feature learning capabilities.

Despite the strong performance of most existing face anti-spoofing algorithms within their training databases, they often exhibit poor performance in cross-database scenarios. This discrepancy is primarily due to domain shifts caused by variations in capture devices, environmental lighting, and the ethnic diversity of subjects across different databases. Additionally, the increasing prevalence of makeup, driven by improving living standards and the pursuit of beauty, further complicates this issue. Makeup alters facial textures and colors in certain areas, contributing to domain shifts in real-world scenarios, which can interfere with the accuracy of face anti-spoofing systems. Most people wear light makeup in their daily lives, which does not significantly alter their identity features. However, if such cases are incorrectly identified as fraudulent, it can cause significant inconvenience to users. Current research in face recognition and face anti-spoofing pays limited attention to light makeup faces, despite their commonality. This paper focuses on light makeup in daily life and does not address heavy makeup used on special occasions.

Ueda et al. [6] constructed a database comprising bare faces, light makeup faces, and heavy makeup faces to investigate the impact of makeup on face recognition systems. Their experiments revealed that light makeup faces were the easiest to recognize, followed by bare faces and heavy makeup faces. The study concluded that light makeup enhances facial distinctiveness through moderate alterations to facial features, while heavy makeup reduces facial distinctiveness, making it less suitable for face recognition. Chen et al. [7] were the first to examine makeup as a means of fraudulent attack, evaluating the vulnerability of face recognition systems to such attacks. Subsequently, multiple studies [8,9,10,11,12] have shown that attackers can use heavy makeup to mimic the identity of a target person or conceal their own identity, thereby compromising face recognition systems. However, most existing research focuses on heavy makeup as a means of fraudulent attack, and there is a lack of studies on general detection algorithms for light makeup faces. Given that most people in daily life wear light makeup, which does not significantly alter their identity features, misidentification as fraudulent behavior by face anti-spoofing systems can cause significant inconvenience to users. Therefore, this paper does not address heavy makeup used on special occasions but focuses on light makeup in daily life, investigating the generalization of face anti-spoofing algorithms for light makeup faces.

The primary contributions of this paper are outlined as follows:

- We developed a face anti-spoofing database that specifically includes faces with light makeup. To ensure the relevance and quality of the data, we introduced a criterion for determining light makeup during the data selection process. This database represents a novel resource for assessing the impact of light makeup on face anti-spoofing algorithms.
- We conducted evaluations of several existing, representative face anti-spoofing algorithms using the constructed database. The experimental results indicate that the performance of most algorithms declines when processing faces with light makeup. This finding underscores the necessity of developing specialized approaches tailored to this context.
- We propose a general face anti-spoofing algorithm designed for faces with light makeup. This algorithm demonstrates robust generalization capabilities and achieves superior detection performance, particularly when applied to faces with light makeup.

The structure of this paper is organized as follows: Section 2 provides a review of related works on face anti-spoofing. In Section 3, a face anti-spoofing database featuring faces with light makeup is introduced, and the performance of several algorithms in light makeup scenarios is evaluated. Section 4 presents the proposed method, and Section 5 presents an analysis of the experimental results. Finally, Section 6 concludes the paper.

## 2. Related Works

This section categorizes and briefly introduces the face anti-spoofing algorithms under evaluation, facilitating a comparative analysis of experimental results across different algorithms in the subsequent sections. The algorithms are classified into three main types: large model-based methods, cross-domain methods, and binary supervision-based methods.

### 2.1. Large Model-Based Methods

Methods leveraging large models benefit from their strong feature extraction capabilities, typically surpassing other approaches. To facilitate comparative analysis of experimental results among similar algorithms, this section categorizes methods based on large models. ViTAF* (Vision Transformer with Ensemble Adapters and Feature-wise Transformation Layers) [13] utilizes ViT as its backbone, incorporating ensemble adapters and feature-wise transformation layers to adapt each transformer block and perform feature-level data augmentation. This allows for general few-shot cross-domain tasks by learning from balanced source-domain data and limited target-domain samples. FLIP (Cross-domain Face Anti-spoofing with Language Guidance) [14], grounded in CLIP (Contrastive Language-Image Pre-training) [15], proposes three variants—FLIP-V, FLIP-IT, and FLIP-MCL—each with distinct fine-tuning strategies and loss functions. FLIP-V focuses solely on fine tuning the image encoder of CLIP without text representation. FLIP-IT enhances FLIP-V by incorporating text representations for real/fake category language supervision, using the cosine similarity between image and category text representations for predictions. FLIP-MCL builds upon FLIP-IT by applying simCLR [16] and MSE losses to further improve the consistency between image and text representations.

### 2.2. Cross-Domain Methods

Cross-domain methods go beyond using real/fake labels by also considering data-domain or spoofing attack-type labels, aiming to enhance model generalizability through the learning of domain-invariant features or classifiers. SSAN (Shuffled Style Assembly Network) [17] disentangles and recombines content and style features, employing contrastive learning to highlight style information pertinent to real/fake discrimination while minimizing the influence of domain-specific style information. This approach offers two variants: SSAN-R, which uses ResNet-18 [18] as its backbone and predicts based on the output of the sigmoid function for the real class, and SSAN-M, which adopts DepthNet [19] and bases its prediction on the mean of the predicted depth map. SA-FAS (FAS Strategy of Separability and Alignment) [20] promotes the separation of features across different data domains and real/fake categories through supervised contrastive learning. It optimizes the real/fake classification hyperplanes for each domain to align and converge into a global real/fake classification hyperplane, thus achieving a domain-invariant classifier. DGUA-FAS (Domain-Generalized Face Anti-Spoofing with Unknown Attacks) [21] integrates a transformer-based feature extractor with an unknown attack sample generator. It categorizes known samples by their real or spoofing attack type, simulates more dispersed unknown attack samples in the feature space, and generates smooth labels proportional to the input’s real and spoofing types to guide the unknown attack samples. This aids in training the feature extractor to identify domain-invariant real/fake discriminative features. GAC-FAS (Gradient Alignment for Cross-Domain Face Anti-Spoofing) [22] introduces cross-domain gradient alignment, identifying the gradient ascent points of each domain and adjusting the generalized gradient updates to maintain consistency with ERM gradient updates. This encourages the model to converge to the flattest minimum, thereby obtaining a domain-invariant classifier.

### 2.3. Binary Supervision-Based Methods

Binary supervision-based methods differ from the aforementioned cross-domain approaches by relying exclusively on real/fake labels, emphasizing the mining of features from individual samples. LCFF (LBP and CNN Feature Fusion) [23] combines RGB color features with LBP texture features, using a relatively simple CNN for feature extraction and fusion, effectively streamlining the network architecture. IADG (Instance-Aware Domain Generalization Framework) [24] mitigates the sensitivity of features to the specific styles of individual samples by implementing asymmetric sample-adaptive whitening at the granular level of individual samples, promoting the learning of domain-invariant real/fake discriminative features. LDA (Latent Distribution Adjusting) [25] captures the data distribution in each category through multiple prototype centers and implicitly determines a flexible number of centers per class in the final fully connected layer. It classifies based on the prototype centers to achieve binary classification, thereby enhancing the model’s generalization capability by better representing the complex data distribution in the field of face anti-spoofing.

## 3. Construction of a Face Anti-Spoofing Database with Light Makeup Faces

### 3.1. Criterion for Determining Light Makeup

To date, no studies have introduced a quantitative method for establishing the criteria for light makeup. Instead, assessments are predominantly based on subjective evaluations by participants, who rely on specific features. However, these subjective, experience-based judgments are frequently affected by personal biases, resulting in a lack of precision, repeatability, and general applicability.

**Figure 1:**
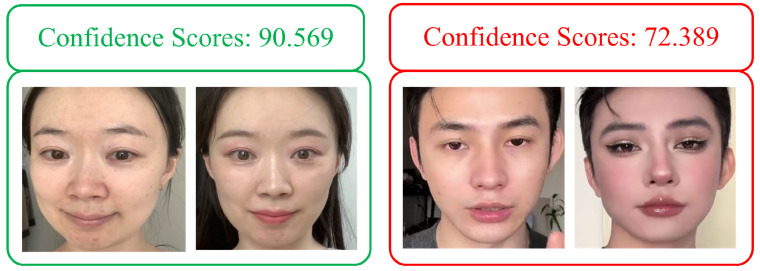
Confidence scores between input bare-faced and makeup face images.

This section proposes a quantitative criterion for determining light makeup to objectively measure the degree of makeup. Drawing on the research by Chen et al. [7], bare-faced and makeup face images of the same identity are input into the Face++ [26] face comparison model. The model outputs confidence scores, which are illustrated in Figure 1, along with the input images. A higher confidence score indicates a greater probability that the model considers the two face images to belong to the same identity. This confidence score serves as the first criterion for determining light makeup, denoted as $LI_{1}$, as shown in Equation (1).

(1) $$LI_{1}=\Phi \left(MK,NMK_{i}\right)$$

where i∈{1,2} denotes the index of the bare-faced image, MK represents the makeup face image, $NMK_{i}$ represents the bare-faced image, and Φ(x,y) is the confidence score output by the face comparison model, indicating the likelihood that the two face images belong to the same identity. A higher $LI_{1}$ value suggests lighter makeup.

The developers of the Face++ face comparison model provide a threshold for determining whether two face images are captured from the same identity. However, this threshold is primarily intended to judge whether two images belong to the same individual, considering factors such as environmental lighting, face angle, and facial expressions, in addition to makeup. Consequently, directly using this threshold as a criterion for light makeup is not accurate. To address this limitation and enhance the generalizability of the light makeup determination criterion, our laboratory collected an image dataset for makeup assessment from the Internet. We conducted statistical analysis on the output confidence scores of makeup and bare-faced images. The dataset, primarily sourced from Little Red Book [27], includes 50 subjects, each with two bare-faced images captured under different environments—one image subjectively judged as light makeup and one image subjectively judged as heavy makeup.

**Figure 2:**
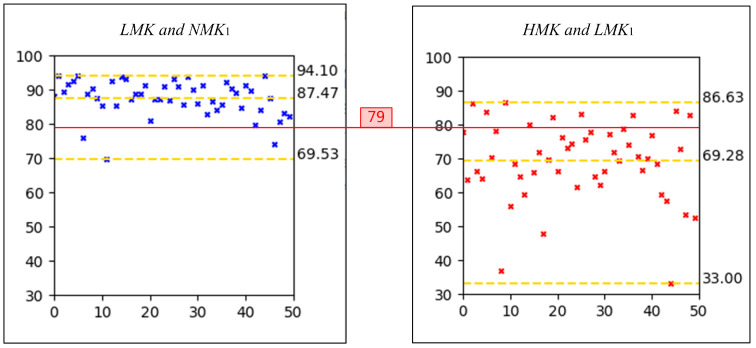
Scatter plots of the $LI_{1}$ values for light makeup and heavy makeup faces.

To determine the $LI_{1}$ threshold, we conducted a statistical analysis of the $LI_{1}$ values for light makeup and heavy makeup faces. Specifically, we input pairs of $NMK_{1}$ and LMK, as well as $NMK_{1}$ and HMK, from the same subject into the face comparison model. The confidence scores were then plotted as scatter plots, as illustrated in Figure 2. In these plots, the x-axis represents the subject’s identity, while the y-axis denotes the confidence score output by the model. The yellow dashed lines in each plot indicate the maximum, mean, and minimum confidence scores. By averaging the $LI_{1}$ values for all image pairs, we obtained a mean value of 78.425. Although enhancing user experience and avoiding inconveniencing innocent users are important considerations, the primary objective of face anti-spoofing is to resist spoofing attacks. To simplify the data and minimize errors, we rounded 78.425 up to 79. Consequently, we established the first criterion for light makeup as $LI_{1}>79$ for both comparisons of the makeup image with the two bare-faced images.

In order to mitigate the effects of environmental lighting, face angle, and facial expression, this section introduces a second criterion for determining light makeup, as represented by Equation (2):

(2) $$LI_{2}=\frac{1}{2}\sum_{i=1}^{2}\left(\Phi \left(NMK_{1},NMK_{2}\right)-\Phi \left(MK,NMK_{i}\right)\right)$$

where the term $\Phi (NMK_{1},NMK_{2})$ within $LI_{2}$ quantifies the confidence score changes attributable to factors other than makeup, thereby reducing the influence of environmental lighting, face angle, and facial expression. A smaller $LI_{2}$ value indicates lighter makeup.

**Figure 3:**
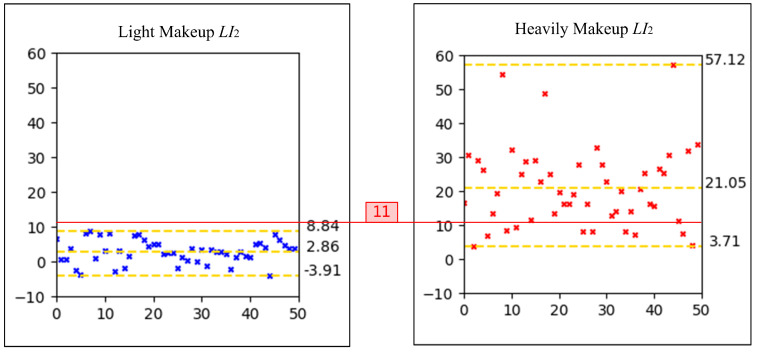
Scatter plots of the $LI_{2}$ values for light makeup and heavy makeup images.

**Figure 4:**
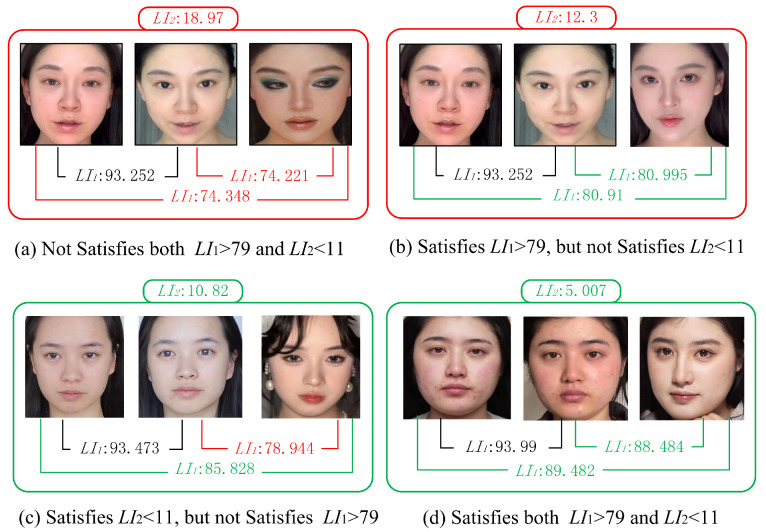
Examples of the judgment, where the first two images in each group are bare-faced images and the third image is the makeup face to be evaluated.

To establish a reasonable threshold for $LI_{2}$, scatter plots of $LI_{2}$ values for light makeup and heavy makeup images from the makeup assessment dataset were generated, as depicted in Figure 3. The y-axis of the scatter plot represents the $LI_{2}$ values. Similar to $LI_{1}$, the average of all image pairs’ $LI_{2}$ values was calculated and rounded down to 11. Consequently, the second criterion for light makeup is set as $LI_{2}<11$. A makeup face image is determined to be light makeup if it satisfies both $LI_{1}>79$ and $LI_{2}<11$. Examples of this judgment are illustrated in Figure 4, where the first two images in each group are bare-faced images and the third image is the makeup face to be evaluated.

Drawing on the approach proposed by Dantcheva et al. [28], this paper generates makeup face images through virtual makeup. The process involves several steps to ensure logical accuracy and detail. First, makeup transfer is performed on the real, bare-faced real images from an existing face anti-spoofing database to create makeup face images. Next, light makeup face images are screened from the generated makeup face images using specific light makeup determination criteria. Finally, these light makeup face images are combined with the original database to form a face anti-spoofing database that includes light makeup faces. The advantages of this approach are twofold:

- It minimizes the differences between bare-faced and makeup faces caused by pose, lighting, and expression, thereby allowing for a focused analysis of the effects of makeup.
- By leveraging an existing face anti-spoofing database, which already contains real, bare-faced images and spoofed face images, the makeup transfer on real, bare-faced images facilitates the construction of a face anti-spoofing database that includes light makeup faces.

### 3.2. Collection of Data for Makeup Transfer

Makeup transfer requires the collection of real, bare-faced images and reference makeup face images. The specific sources are as follows: Real, bare-faced images were primarily obtained from commonly used face anti-spoofing databases, including the MSU-MFSD database [29], Replay-Attack [30], CASIA-FASD [31], and OULU-NPU [32] (hereafter referred to as the M database, R database, C database, and O database, respectively), which contain real face images. Reference makeup face images were mainly collected manually from the Internet, with Little Red Book [27] being the primary source. To ensure high-quality images, only those uploaded by the subjects themselves or makeup artists, taken with the original camera and not post-processed, were collected. This minimizes quality loss and interference during transmission and post-processing. Additionally, the collected images must have a face region occupying at least half of the image, and the facial skin texture should be clearly visible to provide sufficient information and detail for subsequent makeup transfer.

**Figure 5:**
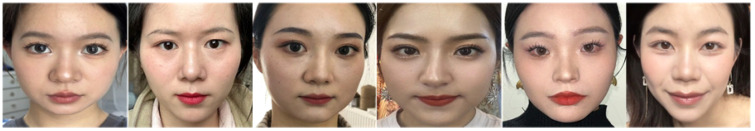
Example reference makeup face images.

Ultimately, a total of 496 reference makeup face images were collected from over 300 individuals. The reference makeup face image dataset was divided according to the ratio of real video counts in the training, validation, and test sets of the four selected face anti-spoofing databases. Example reference makeup face images are shown in Figure 5.

### 3.3. Generating Light Makeup Faces with Makeup Transfer Algorithms

To enrich the makeup effects, this paper employs two makeup transfer algorithms: SpMT (Semi-Parametric Makeup Transfer via Semantic-Aware Correspondence) [33] and EleGANt (Exquisite and Locally Editable GAN for Makeup Transfer) [34]. The SpMT algorithm generates more subtle makeup effects, while the EleGANt algorithm performs better for fine details such as eye makeup, resulting in more noticeable makeup effects.

**Figure 6:**
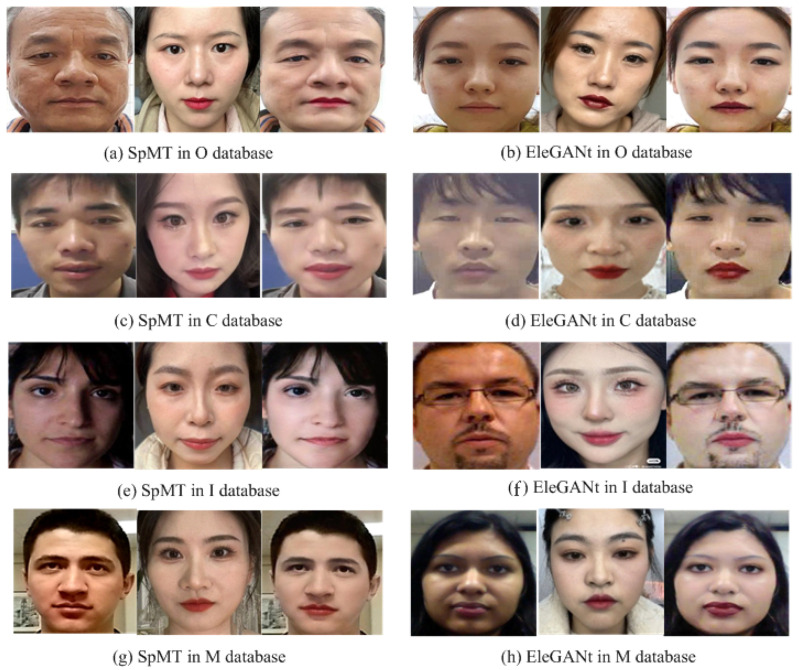
Example triplet of the original real, bare-faced image, the reference makeup face image, and the generated light makeup face image.

This section uses the makeup transfer models released by the original authors of SpMT and EleGANt. The algorithms are applied to the real, bare-faced images from the M database, R database, C database, and O database, using the reference makeup face images as references. For each video in the training, validation, and test sets, a reference makeup face image is randomly selected from the corresponding reference makeup face image set for makeup transfer. To avoid confusion and facilitate subsequent applications, the makeup face images generated using different makeup transfer methods and from different original videos are stored in separate video folders. Next, the real, bare-faced images and the generated makeup face images from the same identity are input into the face comparison system. If the generated makeup face images satisfy the light makeup determination criteria (both $LI_{1}>79$ and $LI_{2}<11$), they are considered light makeup face images and are retained. Figure 6 shows an example triplet of the original real, bare-faced image, the reference makeup face image, and the generated light makeup face image.

To evaluate the performance degradation of algorithms when handling real faces transitioning from bare-faced to light makeup in the target domain, this section replaces the real, bare-faced images in the original face anti-spoofing databases with their corresponding light makeup face images. Additionally, to ensure that the final constructed database includes the shooting environments and identities of all original real, bare-faced videos and contains light makeup videos generated by both makeup transfer methods, this section alternates between the light makeup videos generated by the two methods. If there is no corresponding light makeup video for an original real video, the original real video is used directly.

Ultimately, a face anti-spoofing database was constructed that includes the original bare-faced real videos and light makeup videos generated by both makeup transfer methods (Makeup_Mix, hereafter referred to as Mkx). The specific distribution of the videos is shown in Table 1.

### 3.4. Assessment of Face Anti-Spoofing Algorithms in Light Makeup Scenarios

This section evaluates the performance of existing representative face anti-spoofing algorithms in light makeup scenarios and validates the constructed database by referencing several papers discussed in Section 2.

We evaluate the proposed models using several datasets: the I, C, M, O, and Mkx datasets, which specifically contain spoofing detection data with light makeup. The Mkx dataset is further divided into subsets by transferring makeup from the I, C, M, and O datasets, labeled as Mk(I), Mk(C), Mk(M), and Mk(O), respectively.

The models are initially trained on a source domain comprising faces without light makeup and subsequently tested on two distinct target domains: one primarily consisting of real faces with light makeup and the other with bare faces. This experimental setup facilitates a comparative analysis of the models’ performance in scenarios both with and without light makeup. A leave-one-out testing strategy, a prevalent method in the face anti-spoofing field, is employed. Specifically, the models are trained on the I, C, and M datasets and tested on the O dataset, denoted as ICM_O. Additional testing strategies include OCI_M, OCI_Mk(M), OIM_C, OIM_Mk(C), OCM_I, OCM_Mk(I), and ICM_Mk(O), resulting in a total of eight testing strategies.

The models’ performance is assessed using two widely recognized metrics in the face anti-spoofing domain: Area Under the Curve (AUC) and Half Total Error Rate (HTER). Given that HTER calculation involves threshold selection, this paper adheres to the experimental settings outlined in the SA-FAS and SSAN papers to ensure fair evaluation. Specifically, the threshold is determined at the point on the ROC curve where the value of (TPR-FRR) is maximized. HTER is then calculated using this threshold, and the model with the highest (AUC-HTER) value is identified as the best-performing model. To guarantee the accuracy and reliability of the experimental results, all parameter settings for the evaluated algorithms are sourced from their respective original papers. The specific evaluation of the experimental results is shown in Table 2, Table 3, Table 4 and Table 5.

The results presented in Table 3 exhibit several noteworthy characteristics:

(1) Impact of Light Makeup: The transition of real faces in the target domain from bare to light makeup results in a performance decline for most algorithms. This observation underscores the inadequacy of current face anti-spoofing methods in handling scenarios involving light makeup.

(2) Performance in Zero-shot Scenario: In the zero-shot scenario, cross-domain methods exhibit superior performance, followed by large model-based methods. In contrast, binary supervision-based methods demonstrate relatively poor performance.

(3) Performance Differences Across Testing Strategies: A detailed analysis of the results from various testing strategies indicates that all algorithms experience a significant performance drop in ICM_Mk(O) compared to ICM_O. Conversely, some algorithms show a smaller performance drop in OCI_Mk(M), OIM_Mk(C), and OCM_Mk(I) compared to OCI_M, OIM_C, and OCM_I, with a few algorithms even showing performance improvements.

**Figure 7:**
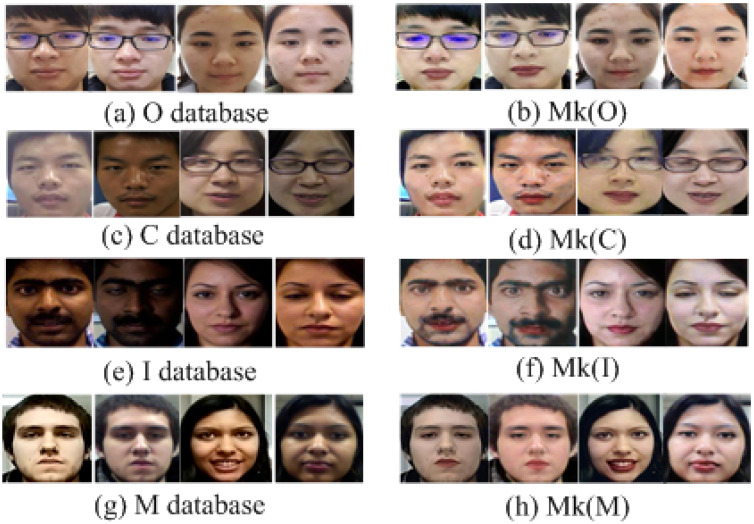
Before-and-after samples of every database makeup transfer.

These differences are primarily attributed to the domain variations within the O, C, M, and I datasets, as well as the reference makeup images used for makeup transfer, as depicted in Figure 7. The O dataset was primarily collected from domestic identities with minimal variations in lighting conditions. In contrast, the C dataset, also from domestic identities, exhibits larger variations in lighting conditions. The I and M datasets are predominantly composed of foreign identities, characterized by significant population distribution differences and greater variations in lighting conditions. For makeup transfer, high-quality reference makeup images are mainly sourced from makeup display photos of domestic women, as shown in Figure 5, which exhibit smaller variations in population distribution and lighting conditions. Makeup transfer aims to maintain the original lighting conditions and identity features of the bare face images. However, the process inherently modifies skin tone and facial features. Consequently, makeup transfer typically involves minor adjustments to the bare face images based on the reference makeup images, leading to reduced domain variations in population distribution and lighting conditions compared to the original datasets. Nevertheless, makeup transfer also introduces domain variations in makeup. The varying generalization abilities of different algorithms to these two types of variations—population distribution and lighting conditions—result in differing performance across various testing strategies.

## 4. The Proposed Method

### 4.1. Overview

**Figure 8:**
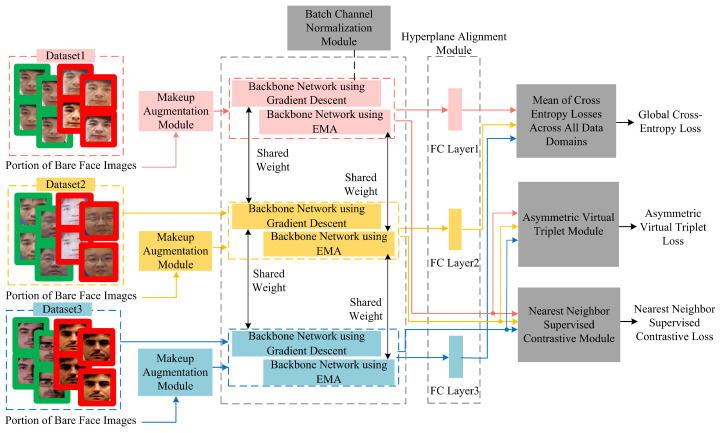
Framework of the proposed method.

Current face anti-spoofing methods often exhibit performance limitations when applied to light makeup faces, as highlighted by prior research. To address this challenge, this paper introduces a general face anti-spoofing algorithm specifically tailored for light makeup faces. The proposed algorithm extends the SA-FAS framework by incorporating several innovative components: a makeup augmentation module, a batch channel normalization module, a backbone network updated via the Exponential Moving Average (EMA) method, an asymmetric virtual triplet loss module, and a nearest neighbor supervised contrastive module. These enhancements collectively form the advanced architecture illustrated in Figure 8. By refining the algorithm’s design, this paper aims to enhance clarity and engagement while maintaining the technical precision and objectivity necessary for submission to a top-tier journal or conference.

The overall workflow of the proposed algorithm during training is as follows: Initially, makeup images are selected as the reference set and preprocessed alongside the training dataset, including the extraction of facial landmarks. Subsequently, a portion of genuine samples from the preprocessed training data is fed into the makeup augmentation module for cosmetic augmentation. Both the original images from the training set that have not undergone makeup augmentation and the augmented images are then input into the backbone network, which is updated using both gradient descent and the exponential moving average (EMA) method. This process extracts the features denoted as $F_{\mathrm{grad}}$ and $F_{\mathrm{EMA}}$, where the backbone network is an improved ResNet-18 model enhanced with a batch channel normalization module.

$F_{\mathrm{grad}}$ is subsequently fed into the hyperplane alignment module for each data domain, training binary classifiers for each domain to obtain the optimal real–fake classification hyperplanes. These hyperplanes are optimized and converged into a global real–fake classification hyperplane, and the mean of the cross-entropy losses across all data domains is calculated to derive the global cross-entropy loss. Meanwhile, $F_{\mathrm{grad}}$ and $F_{\mathrm{EMA}}$ are input into the nearest neighbor supervised contrastive module to compute the nearest neighbor supervised contrastive loss. Additionally, $F_{\mathrm{grad}}$ is input into the asymmetric virtual triplet module, forming an asymmetric virtual triplet together with two fixed orthogonal vectors, to calculate the asymmetric virtual triplet loss. The combination of the global cross-entropy loss, the nearest neighbor supervised contrastive loss, and the asymmetric virtual triplet loss serves as the composite loss function to supervise the training of the network model.

During testing, the test dataset is input into the backbone network updated using the gradient descent method, outputting binary real–fake predictions. The following sections provide detailed descriptions of each module’s specific structure.

### 4.2. Makeup Augmentation Module

**Figure 9:**
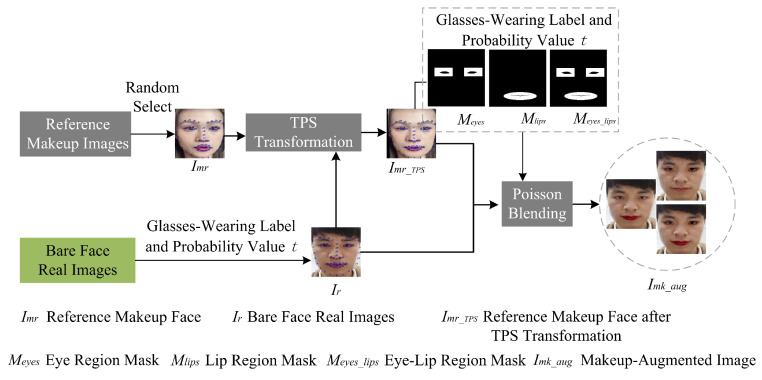
Structural diagram of the makeup augmentation module.

The specific results of the module are illustrated in Figure 9. To enhance computational efficiency and reduce the number of parameters, the module is designed to perform a simple “makeup application” and is not equipped to handle complex scenarios. First, reference makeup images are selected from a public makeup face image database. However, there are certain limitations when selecting these reference images. For instance, if the reference makeup face image is a large-angle profile, parts of the mouth or eyes may be missing, and the makeup augmentation module does not have the capability to complete these missing parts. Additionally, if the reference makeup face image shows a widely open mouth, Thin Plate Spline (TPS) transformation may distort the shape of the teeth, introducing artifacts. Furthermore, if hair obstructs the selected eye and lip regions, the hair in these areas will be merged with the original bare face during Poisson blending.

**Figure 10:**
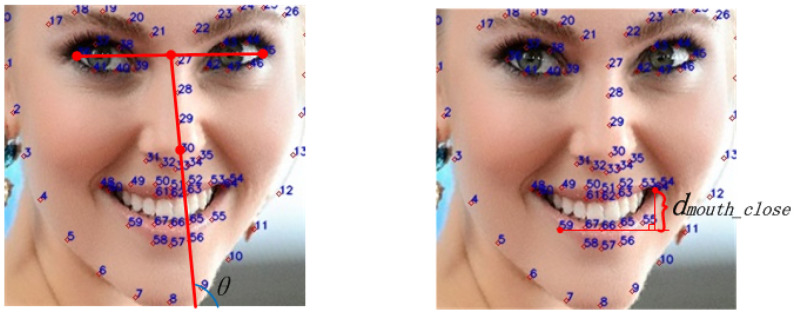
Sketch map of the reference makeup image screening standard.

To address the aforementioned issues and reduce artifacts in makeup augmentation results, this section employs 68 facial landmarks extracted using Dlib to screen images for specific criteria: frontal face orientation, closed-mouth position, and unobstructed eye and lip regions. The screening process is as follows. Frontal Face Orientation: As illustrated in the left image of Figure 10, the midpoint between the outer corners of the left eye (landmark 36) and the right eye (landmark 45) is connected to the tip of the nose (landmark 30). The angle (θ) formed between this line and the horizontal axis at the bottom of the image is measured. If θ falls within the range of $\theta_{1}$ to $\theta_{2}$$\left(\theta_{1}<90^{\circ }<\theta_{2}\right)$, the image is classified as a frontal face. Closed-Mouth Position: As depicted in the right image of Figure 10, the vertical distance between the highest and lowest internal mouth landmarks (landmarks 59–67) is calculated. If this distance is less than a predefined threshold ($d_{\mathrm{mouth}\text{\_}\mathrm{close}}$), the image is considered to have a closed mouth. No Hair Obstruction: It is essential that hair does not obstruct the eye and lip regions, as these areas are selected for Poisson blending. The specific ranges for these regions are detailed in subsequent sections. By adhering to these criteria, the methodology ensures the selection of images that are optimal for makeup augmentation, thereby minimizing potential artifacts.

To address the introduction of light makeup domain shifts while preserving the inherent characteristics of bare-faced real images, a subset of these images is selected from the training data. Eye, lip, and eye–lip makeup augmentations are then applied to a portion of this subset. It is important to note that individuals wearing glasses in bare-faced real images are susceptible to artifacts during eye makeup augmentation. Consequently, eye makeup augmentation is exclusively performed on bare-faced real images of individuals who are not wearing glasses.

To determine whether an input image is a real face based on the true/false label, a random probability value (*t*) is generated, where 0<t<1. Based on the glasses-wearing label and the probability value (*t*), the following decisions are made regarding whether to apply makeup augmentation and the specific regions to augment: If the image is a real face and $t<T_{1}$, lip makeup augmentation is applied to the bare-faced real image. If the image is a real face of a person not wearing glasses and $T_{1}\leq t<T_{2}$, both lip and eye makeup augmentation are applied to the bare-faced real image. If the image is a real face of a person not wearing glasses and $T_{2}\leq t<T_{3}$, eye makeup augmentation is applied to the bare-faced real image.

**Figure 11:**
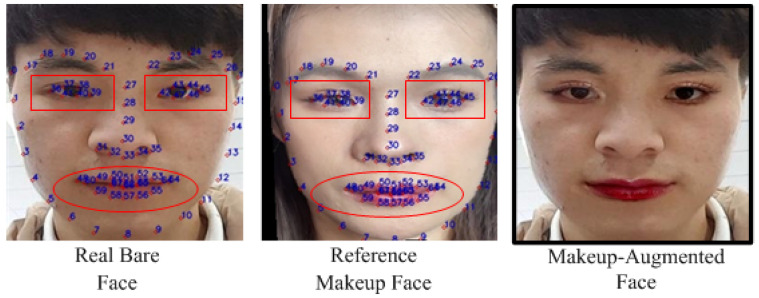
Sketch map of the Poisson fusion area.

For each bare-faced real image ($I_{r}$) selected for makeup augmentation, a reference makeup face image ($I_{mr}$) is randomly chosen from the reference makeup face image set. The makeup augmentation process involves the following steps:

(1) TPS Transformation: Thin plate spline (TPS) transformation is performed on $I_{mr}$ using the facial landmarks of $I_{r}$ as reference points and the facial landmarks of $I_{mr}$ as control points. Affine transformation is used to map the control points to the positions of the reference points. The TPS interpolation formula is applied to correct other pixel points, using the transformed control point coordinates in the TPS interpolation function. This results in the TPS-transformed reference makeup face image ($I_{mr_{-TPS}}$).

(2) Poisson Blending Mask Generation: A mask is generated for the Poisson blending region based on the facial landmarks of $I_{mr_{-TPS}}$. To maximize the makeup effect while preserving the identity features of the bare-faced real image and reducing the quality requirements for the reference makeup face image, smaller eye and lip regions are selected, as shown in Figure 11. The minimum bounding box is computed for the left eye landmarks in $I_{mr_{-TPS}}$. Keeping the center of the bounding box unchanged, the bounding box is expanded to twice its original size. The left eye connection points are removed from this expanded region to obtain the left eye periphery region. The right eye periphery region is selected similarly. The minimum bounding box is computed for the mouth landmarks in $I_{mr_{-TPS}}$. The center of this bounding box is used as the center of an ellipse, and the length and width of the bounding box are used as the major and minor axes of the ellipse, respectively. The inner lip connection points are removed from this elliptical region to obtain the lip periphery region. The eye region mask ($M_{\mathrm{eyes}}$) and the lip region mask ($M_{\mathrm{lips}}$) are then generated. These masks are combined using a bitwise OR operation to obtain the eye–lip region mask ($M_{\mathrm{eyes}\text{\_}\mathrm{lips}}$).

(3) Poisson Blending: Based on the probability (*t*) and the glasses-wearing label, one of the masks is selected ($M_{\mathrm{eyes}}$, $M_{\mathrm{lips}}$, or $M_{\mathrm{eyes}\text{\_}\mathrm{lips}}$) corresponding to the designated makeup augmentation region. Poisson blending of $I_{mr_{-TPS}}$ and $I_{r}$ is performed using the selected mask to generate the final makeup-augmented image ($I_{mk_{-aug}}$).

### 4.3. Batch Channel Normalization Module

Traditional batch normalization and channel normalization often demonstrate suboptimal performance with small batch inputs due to their reliance on local information normalization and restricted parameter sharing. These limitations adversely affect the model’s overall performance and its ability to generalize. To address these challenges, this paper proposes the integration of a batch channel normalization module into ResNet18, inspired by the work of Khaled et al. [35]. By replacing the existing batch normalization layers with this module, the study aims to improve the model’s adaptability across diverse datasets, thereby enhancing its performance and generalization capabilities.

**Figure 12:**
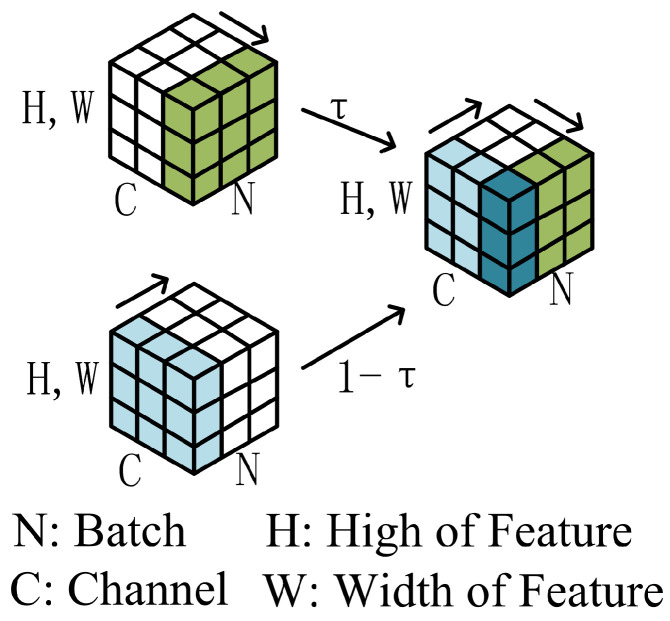
Schematic diagram of a batch channel-normalized module.

As depicted in Figure 12, the batch channel normalization module normalizes the input data across both the channel and batch dimensions. It calculates the mean $\mu_{1}$ and variance ($\sigma_{1}^{2}$) over the (N,H,W) axes and the mean $\mu_{2}$ and variance ($\sigma_{2}^{2}$) over the (C,H,W) axes. Subsequently, the input ($q_{r}$) is normalized using these statistics to produce ${\bar{q}}_{1}$ and ${\bar{q}}_{2}$, as given by the following equations:

(3) $$\begin{array}{cc} {\bar{q}}_{1} & =\frac{\left(q_{r}-\mu_{1}\right)}{\sqrt{\left(\sigma_{1}^{2}+\epsilon \right)}} \end{array}$$

(4) $$\begin{array}{cc} {\bar{q}}_{2} & =\frac{\left(q_{r}-\mu_{2}\right)}{\sqrt{\left(\sigma_{2}^{2}+\epsilon \right)}} \end{array}$$

where $q_{r}$ is the input and ϵ is a small constant to prevent division by zero. The final output (*Y*) is obtained by adaptively weighting the two normalized outputs and applying a linear transformation, as described by the following formula:

(5) $$Y=\gamma (\tau {\bar{q}}_{1}+\left(1-\tau \right){\bar{q}}_{2})+\varphi$$

In this context, *Y* represents the extracted features, while τ, γ, and φ are learnable parameters. Specifically, τ balances the contributions of the normalized outputs along the (N,H,W) and (C,H,W) axes, γ scales the normalized values, and φ shifts the output.

### 4.4. Asymmetric Virtual Triplet Loss Module

In the field of face anti-spoofing, the distribution of samples between genuine and fake categories is often imbalanced. Relying solely on global cross-entropy loss for model training can lead to suboptimal performance for the minority class. Inspired by the virtual triplet loss introduced by Beuve et al. [36], we propose an asymmetric virtual triplet loss.

The virtual triplet loss is an enhancement of the traditional triplet loss. It constructs two fixed orthogonal vectors ($d^{c}$ and $d^{1-c}$) to replace the positive and negative samples in classical triplets, forming virtual triplets with the extracted feature vectors. Here, c∈{0,1} denotes the genuine and fake categories, and the lengths of $d^{c}$ and $d^{1-c}$ match the length of the feature vector extracted from a single sample ($D_{\mathrm{sample}}$). The first half of the components of $d^{c}$ are set to 0, and the second half are set to 1, while the first half of the components of $d^{1-c}$ are set to 1, and the second half are set to 0. For odd lengths, the middle element of both $d^{c}$ and $d^{1-c}$ is set to 0. This approach not only separates genuine and fake samples effectively, similar to the classical triplet loss, but also simplifies the process by avoiding the need to find optimal triplets, thus reducing computational complexity.

In face anti-spoofing, the distribution differences among fake samples are generally larger than those among genuine samples. Therefore, we further improve the virtual triplet loss by introducing an asymmetric virtual triplet loss. This loss treats genuine and fake samples differently, making genuine samples more compact in the feature space and fake samples more dispersed. The formula for the asymmetric virtual triplet loss is the same as that for the virtual triplet loss, as shown in Equation (6):

(6) $$L_{\mathrm{AmdmyT}}\left(F_{z},c\right)=\max\left(2F_{z}\cdot d^{1-c}-2F_{z}\cdot d^{c}+\rho ,0\right)$$

Here, $F_{z}$ represents the feature vector extracted from a single sample, and ρ is the margin of the loss function. Unlike the virtual triplet loss, we set the first $l_{1-c}$ components of $d^{c}$ to 0 and the last $l_{c}$ components to 1, while the first $l_{1-c}$ components of $d^{1-c}$ are set to 1 and the last $l_{c}$ components to 0. If the product of $D_{\mathrm{sample}}$ and $l_{c}$ is not an integer, the fractional part of the product is set to 0 to ensure that $d^{c}$ and $d^{1-c}$ remain orthogonal. Since the extracted features ($F_{z}$) are all positive, when $l_{c}<l_{1-c}$, the asymmetric virtual triplet loss applied to the genuine category is greater than that applied to the fake category, forcing genuine samples to be more compact in the feature space. The values of $l_{c}$ and $l_{1-c}$ are given by

(7) $$l_{c}+l_{1-c}=1,\;0<l_{c}<l_{1-c}<1$$

This design ensures that the model can better handle imbalance in sample distribution and improve the overall performance in face anti-spoofing tasks.

### 4.5. Nearest Neighbor Supervised Contrastive Module

SA-FAS employs supervised contrastive learning [37] to separate features from different data domains, then optimizes the true–fake classification hyperplanes for each domain, aligning them to converge into a global true–fake classification hyperplane. This global hyperplane performs well on both source-domain data and target-domain data. The effectiveness of supervised contrastive learning in separating features from different data domains directly impacts the alignment of the true–fake classification hyperplanes, which is crucial for the training of domain-invariant classifiers.

However, traditional supervised contrastive learning often relies on two views of the same image to construct positive pairs, maximizing the similarity between positive pairs. This approach leads the relationships and distributions among samples being ignored, resulting in less comprehensive and diverse feature representations. Therefore, inspired by Zheng et al. [38], we introduce and improve neighbor-supervised contrastive learning.

**Figure 13:**
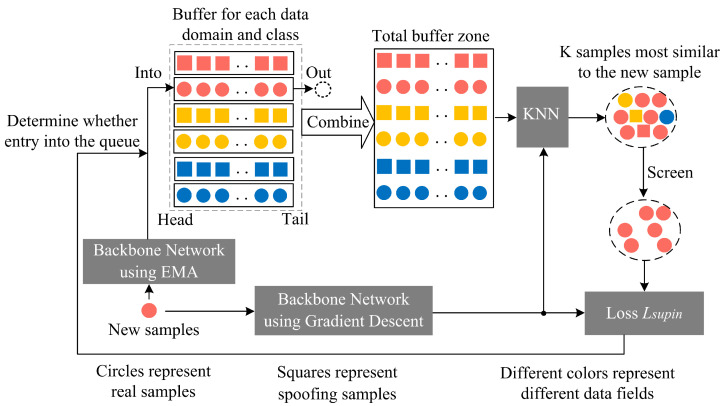
Process of the improved neighbor-supervised contrastive learning.

The specific process of the improved neighbor-supervised contrastive learning is shown in Figure 13. At the beginning of training, $N_{\mathrm{nbuffer}}$ empty buffers are initialized:

(8) $$N_{\mathrm{nbuffer}}=N_{\mathrm{ndomain}}\times N_{\mathrm{nclasses}}+1$$

where $N_{\mathrm{ndomain}}$ is the number of data domains and $N_{\mathrm{nclasses}}$ is the number of classes. In the context of face anti-spoofing, $N_{\mathrm{nclasses}}=2$. The first $N_{\mathrm{ndomain}}\times N_{\mathrm{nclasses}}$ buffers are for the feature caches of each data domain and class, capable of storing up to $N_{\mathrm{buffer}}/\left(N_{\mathrm{ndomain}}\times N_{\mathrm{nclasses}}\right)$ sample features. The last buffer is the global cache, capable of storing up to $N_{\mathrm{buffer}}$ sample features. Each buffer is a queue data structure, where samples enter from the tail and leave from the head.

During training, to stabilize the sample features in the buffers, we use an exponential moving average (EMA) updated backbone network ($\Phi_{\mathrm{EMA}}$) to extract features from new samples. The specific update formula is

(9) $$\Phi_{\mathrm{EMA}}\leftarrow \vartheta \Phi_{{\mathrm{EMA}}_{\mathrm{old}}}+\left(1-\vartheta \right)\Phi_{\mathrm{grad}}$$

where $\Phi_{{\mathrm{EMA}}_{\mathrm{old}}}$ is the EMA-updated backbone network from the previous update, $\Phi_{\mathrm{grad}}$ is the backbone network updated by gradient descent, and ϑ is the momentum coefficient.

When the buffer is not full, the new sample features extracted by $\Phi_{\mathrm{EMA}}$ directly enter the corresponding data domain and class buffer from the tail. When the buffer is full, the new sample enters the buffer only if its neighbor-supervised contrastive loss is less than that of the last sample that entered the buffer, and the oldest sample leaves the buffer. Otherwise, the buffer remains unchanged.

Additionally, we use $\Phi_{\mathrm{grad}}$ to extract features from new samples and compute the dot product with the features in the buffer to obtain a similarity matrix. Based on the KNN algorithm, we select the top *K* most similar samples from the buffer and set the elements in the similarity matrix corresponding to these *K* samples to 0. The selected *K* samples’ similarity matrix is then normalized. According to the true–fake labels and database labels, we select *N* samples from the normalized similarity matrix that belong to the same class and database as the new sample as neighbor samples. The neighbor-supervised contrastive loss ($L_{\mathrm{supin}}$) is computed to determine whether the new sample should enter the buffer and to supervise model training. The specific formula for $L_{\mathrm{supin}}$ is

(10) $$L_{\mathrm{supin}}=-\frac{1}{N}\sum_{u=1}^{N}\log\left(g_{u}\right)$$

where $g_{u}$ is the weight of the neighbor sample in the similarity matrix and *u* is the index of the neighbor sample.

This approach ensures that the model can effectively handle the imbalance in sample distribution and improve the overall performance in face anti-spoofing tasks.

### 4.6. Total Loss Function

Consequently, the total loss function of the algorithm network introduced in this chapter integrates three components: global cross-entropy loss ($L_{\mathrm{cls}}$), asymmetric triplet-like loss ($L_{\mathrm{AmdmyT}}$), and nearest neighbor supervised contrastive loss ($L_{\mathrm{supin}}$). These components are combined into the final loss function (*L*) through a weighted sum:

(11) $$L=\omega_{1}\times L_{\mathrm{cls}}+\omega_{2}\times L_{\mathrm{AmdmyT}}+\omega_{3}\times L_{\mathrm{supin}}$$

where, $\omega_{1}$, $\omega_{2}$, and $\omega_{3}$ denote the respective weights assigned to the three losses. The global cross-entropy loss quantifies the difference between predicted and actual labels, the asymmetric triplet-like loss is designed to strengthen the model’s ability to differentiate between distinct sample categories, and the nearest neighbor supervised contrastive loss serves to refine the model’s performance in distinguishing closely related samples. This integrated loss framework facilitates the optimization of the model during the training phase.

## 5. Experimental Results and Analysis

### 5.1. Experimental Setups

The specific experimental settings and evaluation metrics for this chapter are detailed in Section 4.2. Before training, according to the standards outlined in Section 5.1 for the makeup augmentation module, images from the cropped face region of the MT database were filtered to select frontal faces with closed mouths and no facial occlusion by hair as reference makeup images. The $\theta_{1}$, $\theta_{2}$, and $d_{\mathrm{mouth}\text{\_}\mathrm{close}}$ parameters were set to $80^{\circ }$, $110^{\circ }$, and 5 pixels, respectively, resulting in a total of 173 reference makeup images.

During training, a portion of the real face images from the training set were subjected to makeup augmentation. Both the augmented and non-augmented images underwent standard data augmentation procedures, including random cropping, scaling, random flipping, and normalization. The cropping scale ranged from 20% to 100% of the original image size, maintaining an aspect ratio of 1 for the cropping box. Cropped images were resized to a uniform size of 256×256×3.

For the hyperparameter settings of model training, the $T_{1}$, $T_{2}$, and $T_{3}$ parameters for the makeup augmentation module were set to 0.1, 0.35, and 0.6, respectively. The margin (ρ) for the asymmetric virtual triplet loss function was set to 1, with components $l_{c}$ and $l_{1-c}$ set to 1/3 and 2/3, respectively. In the nearest neighbor supervised contrastive loss module, the maximum number of samples stored in the buffer ($N_{\mathrm{buffer}}$) was set to 1080, the number of nearest neighbors (*K*) in the KNN clustering algorithm was set to 256, and the update momentum coefficient (ϑ) for $\Phi_{\mathrm{EMA}}$ was set to 0.996. In the initial model development, the weighting factors ($\omega_{1}$, $\omega_{2}$, and $\omega_{3}$) for the three losses ($L_{\mathrm{cls}}$, $L_{\mathrm{AmdmyT}}$, and $L_{\mathrm{supin}}$) were all set to 1. This ensures that each component of the loss function contributes equally to the model’s training process. This equal weighting approach helps establish a baseline model that optimally balances all loss components, providing a starting point for further experiments and adjustments.

Training was performed using Stochastic Gradient Descent (SGD) with an initial learning rate of 0.005, a momentum of 0.9, and a weight decay of 0.0005. The learning rate was adjusted every 40 epochs by multiplying the current learning rate by 0.5. The model parameters were iteratively updated based on the combined loss function ($L=L_{\mathrm{cls}}+L_{\mathrm{AmdmyT}}+L_{\mathrm{supin}}$). The training duration was 100 epochs, with a batch size of 90.

After training, the model with the highest AUC-HTER on the validation set was saved. During testing, the best model saved during training was loaded, and seven frames were selected at equal intervals from each video folder in the test set. These frames were input into the test model, and the average prediction score of the seven frames was used as the final classification result for the video.

### 5.2. Comparison with State-of-the-Art Methods

The experimental results of the proposed algorithm in the light makeup face scenario are shown in Table 6. The average results of the four testing strategies of the algorithm presented in this chapter are the best among all methods, with an AUC of 92.21%, which is significantly higher than the second-best result of GAC-FAS, at 90.08%, and an HTER of 13.82%, which is significantly lower than the second-best result of SSAN-M, at 16.75%. Notably, the algorithm achieves the best performance on both the OIM_Mk(C) and ICM_Mk(O) datasets. Specifically, on the ICM_Mk(O) dataset, the AUC reaches 91.16% and the HTER is as low as 15.56%, which is significantly better than the second-best result of GAC-FAS, with an AUC of 89.58% and an HTER of 19.26%. These results clearly demonstrate the generalization ability of the proposed algorithm in detecting light makeup faces.

The experimental results of the proposed algorithm on common public databases without light makeup faces are shown in Table 7. Although the algorithm does not achieve the best performance, it maintains an AUC greater than 96% and an HTER close to or below 10% across all four testing strategies, indicating stable performance. These results further demonstrate the good generalization ability of the proposed algorithm.

### 5.3. Ablation Studies

As shown in Table 8, the addition of various modules significantly enhances the model’s performance in detecting light makeup faces. Specifically, the makeup augmentation module introduces the domain shift caused by light makeup into the source domain as prior knowledge, greatly improving the model’s detection performance in light makeup scenarios. The batch channel normalization module adaptively adjusts the normalization method, enabling the model to better adapt to different datasets. Finally, the asymmetric virtual triplet loss and nearest neighbor supervised contrastive learning modules enhance the separability of sample features in the feature space, thereby improving the generalization performance of the classifier. Therefore, the model demonstrates generalization ability in detecting light makeup faces and exhibits good generalization capabilities.

To assess the impact of weight settings on model performance, we conducted a sensitivity analysis. By varying the values of $\omega_{1}$, $\omega_{2}$, and $\omega_{3}$ while keeping other parameters constant, we observed changes in model performance. The specific experimental results are shown in Table 9, illustrating the variations in AUC and HTER under different weight combinations. We tested various weight combinations, such as (0.5, 0.5, 1), (1, 1, 1), (1, 0.5, 0.5), (0.5, 1, 0.5), and (0.5, 0.5, 0.5), to observe their specific impacts on model performance.

### 5.4. Visualization Analysis

In this section, we employ t-SNE [39] feature visualization to present the experimental results of the proposed algorithm, alongside ablation studies of its two main modules that process the feature space. The ablation experiments include (1) the proposed algorithm without asymmetric virtual triplet loss and (2) the proposed algorithm with nearest neighbor supervised contrastive learning replaced by supervised contrastive learning. By examining the feature space, we aim to investigate the reasons behind the improved performance of the proposed algorithm compared to other methods in the literature. Additionally, we assess the impact of each module on performance enhancement.

**Figure 14:**
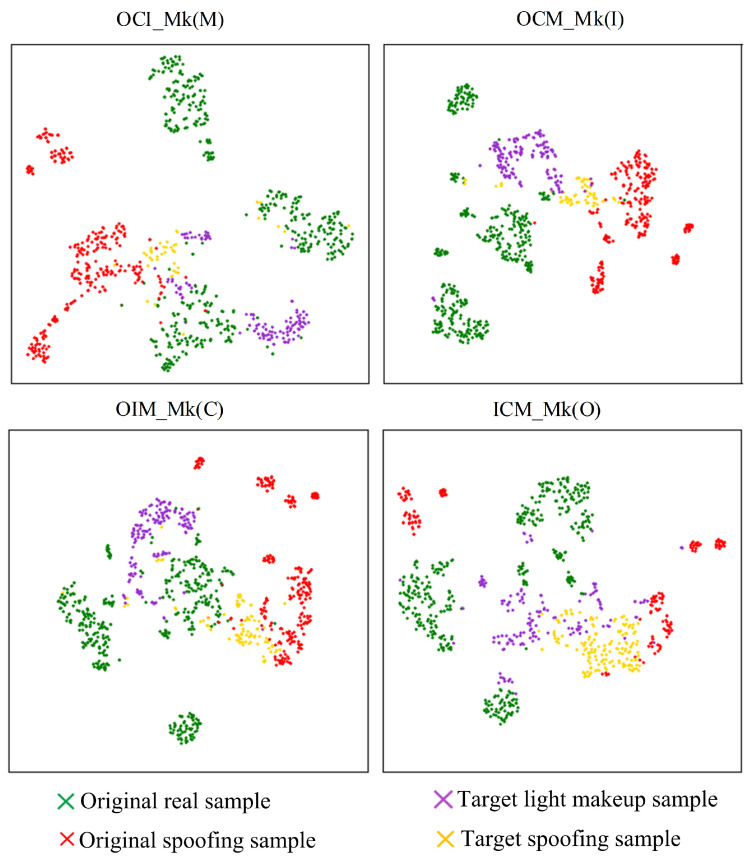
t-SNE visualization of feature separation by the proposed algorithm.

The t-SNE feature visualization of the experimental results of the proposed algorithm is shown in Figure 14. It can be observed that the proposed algorithm significantly reduces the mixing of target-domain spoofing sample features and light makeup sample features, bringing them closer to the corresponding class features in the source domain. Particularly in the OCM_Mk(I) and OIM_Mk(C) testing strategies, the target-domain spoofing sample features and light makeup sample features exhibit minimal mixing and largely overlap with the corresponding class features in the source domain. This effectively avoids the misclassification of light makeup samples and the missed detection of spoofing samples. From the perspective of the feature space, this demonstrates that the proposed algorithm outperforms other methods in the literature in terms of classification performance for light makeup faces and explains the reasons for the performance improvement.

**Figure 15:**
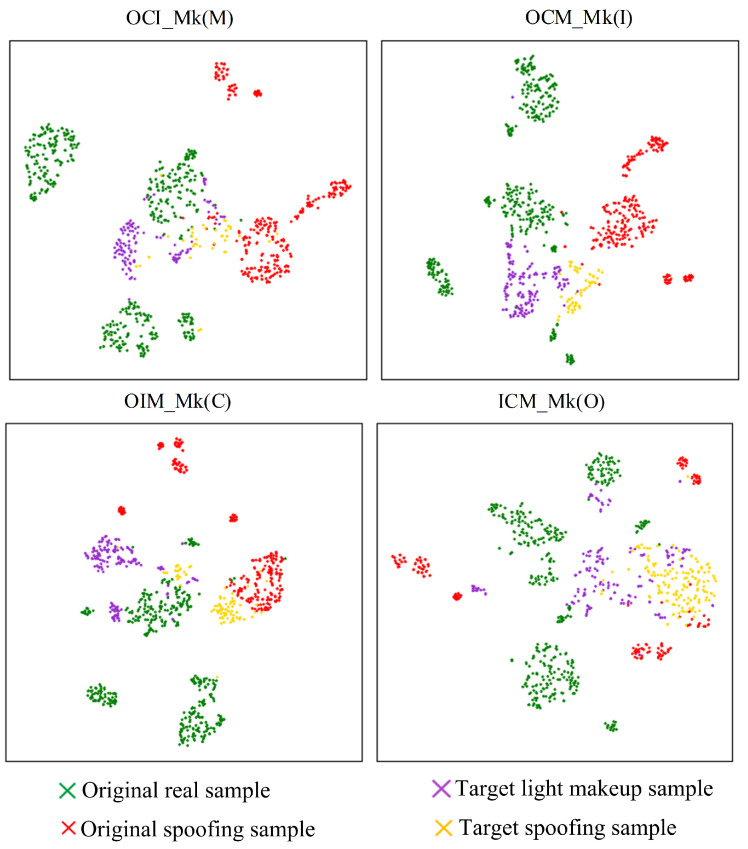
Impact of asymmetric virtual triplet loss on feature separation.

From Figure 14 and Figure 15, it can be observed that after incorporating the asymmetric virtual triplet loss, the mixing of target-domain spoofing sample features and light makeup sample features significantly decreases, and they become closer to the corresponding class features in the source domain, leading to an improvement in classification performance. Particularly in the OCM_Mk(I) and ICM_Mk(O) testing strategies, the incorporation of the asymmetric virtual triplet loss further increases the distance between genuine and spoofing sample features in the source domain while making the clustering of genuine sample features in the source domain more compact. This significantly enhances the separability of genuine and spoofing sample features in the target domain, thereby improving the performance of the model.

**Figure 16:**
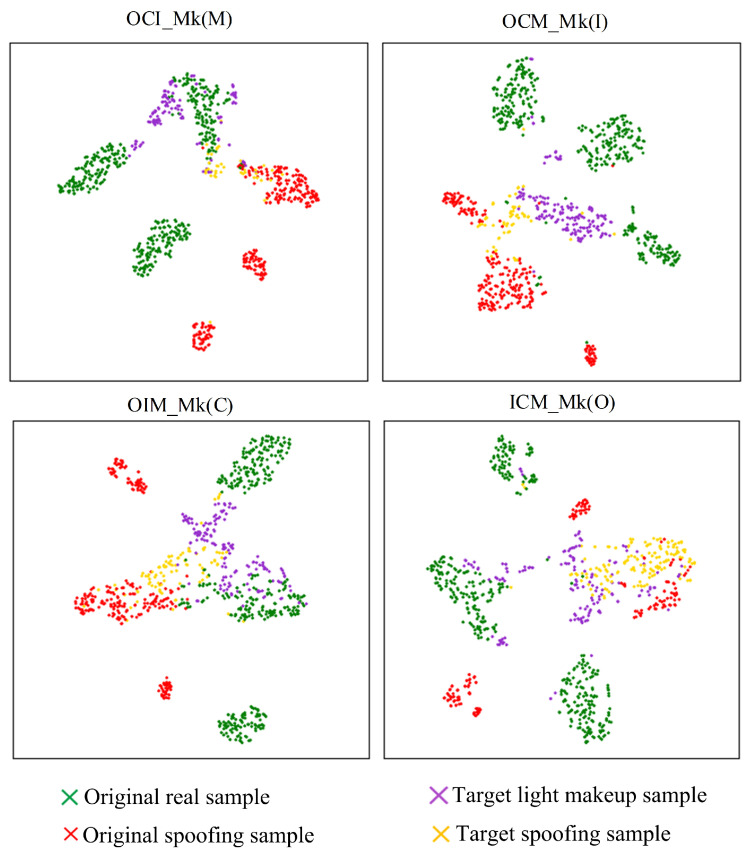
Comparison of supervised contrastive learning vs. nearest neighbor supervised contrastive learning.

Further observation of Figure 14 and Figure 16 reveals that when using supervised contrastive learning, the target-domain spoofing sample features and light makeup sample features exhibit significant mixing. Specifically, in the OCM_Mk(I) and ICM_Mk(O) testing strategies, some light makeup sample features in the target domain mix with the spoofing sample features in the source domain. In the OCI_Mk(M) and OIM_Mk(C) testing strategies, some spoofing sample features in the target domain mix with the genuine sample features in the source domain. These mixings lead to a decrease in classification performance. Compared to supervised contrastive learning, the use of nearest neighbor supervised contrastive learning results in more dispersed clusters of source-domain features. This is because nearest neighbor supervised contrastive learning constructs positive pairs using neighboring samples, which better captures the distribution and relationships of the samples. This approach enhances the separation between different data domains and significantly improves the alignment of the genuine and spoofing classification hyperplanes across different data domains.

## 6. Conclusions

This paper presented the construction of a face anti-spoofing database featuring light makeup faces and introduces a criterion for determining light makeup for screening purposes. We then assessed several existing representative face anti-spoofing algorithms using this newly constructed database. The experimental results indicate that most of these algorithms exhibit reduced performance when confronted with light makeup faces. In response, we proposed a face anti-spoofing algorithm that demonstrates general and strong generalization performance, particularly in detecting light makeup faces. Future research will aim to expand the database by collecting more real-world data of light makeup faces, refine the light makeup determination criteria, and further enhance the algorithm’s generalization capabilities.

Building on the insights gained from this research, we are poised to extend our work in several promising directions: (1) We plan to expand our database to include a broader range of user profiles, such as individuals wearing glasses or hats. This will allow us to better simulate real-world conditions where facial recognition systems must accommodate various accessories without requiring users to remove them. (2) We aim to further refine our algorithms to handle the variability in user presentations more effectively. This includes developing features that are less sensitive to changes in appearance due to makeup or accessories, thereby improving the robustness of facial recognition systems across diverse environments. (3) We will delve deeper into the practical implications of our findings, particularly in settings like TSA security lines, where the ability to accurately recognize individuals wearing various accessories is crucial.

## Figures and Tables

**Table 1:** Number of Light makeup and real bare-faced videos in the MKx database.

Database	M			R			C			O		
	Bare	SpMT	EleGANt	Bare	SpMT	EleGANt	Bare	SpMT	EleGANt	Bare	SpMT	EleGANt
Training Set	2	15	13	1	29	30	0	31	29	1	183	176
Validation Set	-	-	-	0	29	31	-	-	-	0	137	133
Test Set	3	19	18	4	38	38	0	45	44	2	187	171

**Table 2:** Results of cross-database experiments based on large model methods without light makeup in the target domain (%).

Test Strategy Metric		OCM_I		OIM_C		OCI_M		ICM_O	
		**AUC**	**HTER**	**AUC**	**HTER**	**AUC**	**HTER**	**AUC**	**HTER**
0-shot	FLIP-V [14]	98.80	4.71	99.75	1.27	99.31	3.79	98.76	4.15
	FLIP-IT [14]	99.42	2.94	99.98	0.44	98.41	5.24	99.15	3.61
	FLIP-MCL [14]	99.07	4.25	99.98	0.54	98.11	4.95	99.63	2.31
5-shot	ViTAF* [13]	99.64	1.64	99.92	1.40	99.62	2.92	98.67	5.39
	FLIP-V [14]	99.47	1.68	99.84	1.01	99.67	1.89	98.76	4.15
	FLIP-IT [14]	99.83	1.18	99.97	0.46	99.55	2.63	99.30	3.07
	FLIP-MCL [14]	99.86	1.52	99.98	0.63	99.34	3.42	99.81	1.54

**Table 3:** Results of cross-database experiments based on large model methods with light makeup in the target domain (%).

Test Strategy Metric		OCM_Mk(I)		OIM_Mk(C)		OCI_Mk(M)		ICM_Mk(O)	
		**AUC**	**HTER**	**AUC**	**HTER**	**AUC**	**HTER**	**AUC**	**HTER**
0-shot	FLIP-V [14]	86.27	21.89	91.87	15.52	96.29	10.28	84.96	22.59
	FLIP-IT [14]	86.29	20.72	91.30	13.84	95.58	9.03	82.20	22.93
	FLIP-MCL [14]	77.77	26.08	91.12	16.95	91.44	15.00	77.44	>28.33
5-shot	ViTAF* [13]	99.17	4.50	99.85	1.12	99.16	2.50	98.69	5.48
	FLIP-V [14]	98.85	5.06	99.42	3.72	96.42	9.03	97.60	8.73
	FLIP-IT [14]	99.82	1.28	98.14	7.08	99.53	2.50	98.30	6.03
	FLIP-MCL [14]	99.66	1.78	98.19	6.80	99.73	3.06	96.41	9.56

**Table 4:** Results of cross-database experiments based on cross-domain methods and dual supervision methods without light makeup in the target domain (%).

Test Strategy	OCM_I		OIM_C		OCI_M		ICM_O	
**Cross-Domain Methods**	**AUC**	**HTER**	**AUC**	**HTER**	**AUC**	**HTER**	**AUC**	**HTER**
SSAN-R [17]	96.79	8.88	96.67	10.00	98.75	6.67	93.63	13.72
SSAN-M [17]	94.58	14.00	90.81	16.47	94.76	10.42	88.17	19.51
SA-FAS [20]	97.54	6.58	95.37	8.78	96.55	5.95	96.23	10.00
DGUA-FAS [21]	97.75	6.83	96.59	10.06	97.71	7.50	97.34	7.69
GAC-FAS [22]	98.87	4.29	95.16	8.20	97.56	5.00	97.16	8.60
Dual Supervision Methods								
LCFF [23]	81.97	22.36	78.82	25.29	82.56	27.50	83.07	22.59
IADG [24]	94.50	10.62	96.44	8.70	98.19	5.41	97.14	8.86
LDA [25]	90.72	13.50	92.69	12.63	93.02	10.42	93.80	12.22

**Table 5:** Results of cross-database experiments based on cross-domain methods and dual supervision methods with light makeup in the target domain (%).

Test Strategy	OCM_Mk(I)		OIM_Mk(C)		OCI_Mk(M)		ICM_Mk(O)	
**Cross-Domain Methods**	**AUC**	**HTER**	**AUC**	**HTER**	**AUC**	**HTER**	**AUC**	**HTER**
SSAN-R [17]	89.80	19.17	86.91	20.30	95.27	7.92	87.55	21.85
SSAN-M [17]	87.32	19.67	90.08	14.90	93.31	10.42	84.25	21.99
SA-FAS [20]	83.48	23.67	84.64	20.30	93.27	10.42	85.54	21.85
DGUA-FAS [21]	92.68	13.50	82.96	24.58	95.54	10.42	80.64	26.67
GAC-FAS [22]	82.64	22.14	92.21	16.71	95.89	9.23	89.58	19.26
Dual Supervision Methods								
LCFF [23]	85.43	20.33	73.26	33.71	84.81	22.50	79.53	25.93
IADG [24]	80.79	23.08	81.74	22.25	90.63	17.71	86.59	20.19
LDA [25]	87.56	21.67	88.90	18.06	93.48	12.50	78.76	26.94

**Table 6:** Comparison of AUC and HTER with other methods on light makeup faces in the target domain (%).

Test Strategy	OCI_Mk(M)		OCM_Mk(I)		OIM_Mk(C)		ICM_Mk(O)		Average	
	**AUC**	**HTER**	**AUC**	**HTER**	**AUC**	**HTER**	**AUC**	**HTER**	**AUC**	**HTER**
SSAN-R [17]	95.27	7.92	89.80	19.17	86.91	20.30	87.55	21.85	89.88	17.31
SSAN-M [17]	93.31	10.42	87.32	19.67	90.08	14.90	84.25	21.99	88.74	16.75
LCFF [23]	84.81	22.50	85.43	20.33	73.26	33.71	79.53	25.93	80.76	25.62
SA-FAS [20]	93.27	10.42	83.48	23.67	84.64	20.30	85.54	21.85	86.73	19.06
LDA [25]	93.48	12.50	87.56	21.67	88.90	18.06	78.76	26.94	87.18	19.79
IADG [24]	90.63	17.71	80.79	23.08	81.74	22.25	86.59	20.19	84.94	20.81
DGUA-FAS [21]	95.54	10.42	92.68	13.50	82.96	24.58	80.64	26.67	87.96	18.79
GAC-FAS [22]	95.89	9.23	82.64	22.14	92.21	16.71	89.58	19.26	90.08	16.84
Ours	95.27	10.00	91.24	15.00	91.18	14.71	91.16	15.56	92.21	13.82

**Table 7:** Comparison of AUC and HTER with other methods on faces without light makeup in the target domain (%).

Test Strategy	OCI_M		OCM_I		OIM_C		ICM_O		Average	
	**AUC**	**HTER**	**AUC**	**HTER**	**AUC**	**HTER**	**AUC**	**HTER**	**AUC**	**HTER**
SSAN-R [17]	98.75	6.67	96.79	8.88	96.67	10.00	93.63	13.72	96.46	9.82
SSAN-M [17]	94.76	10.42	94.58	14.00	90.81	16.47	88.17	19.51	92.08	15.10
LCFF [23]	82.56	27.50	81.97	22.36	78.82	25.29	83.07	22.59	81.61	24.44
SA-FAS [20]	96.55	5.95	97.54	6.58	95.37	8.78	96.23	10.00	96.42	7.83
LDA [25]	93.02	10.42	90.72	13.50	92.69	12.63	93.80	12.22	92.56	12.19
IADG [24]	98.19	5.41	94.50	10.62	96.44	8.70	97.14	8.86	96.57	8.40
DGUA-FAS [21]	97.71	7.50	97.75	6.83	96.59	10.06	97.34	7.69	97.35	8.02
GAC-FAS [22]	97.56	5.00	98.87	4.29	95.16	8.20	97.16	8.60	97.19	6.52
Ours	97.23	7.92	96.91	10.00	96.20	10.06	96.38	8.89	96.68	9.22

**Table 8:** Comparison of AUC and HTER in ablation experiments (%).

Makeup	Batch Channel	Asymmetric	Contrastive	OCI_Mk(M)		OCM_Mk(I)		OIM_Mk(C)		ICM_Mk(O)	
**Augmentation**	**Normalization**	**Virtual Triplet**	**Learning**	**AUC**	**HTER**	**AUC**	**HTER**	**AUC**	**HTER**	**AUC**	**HTER**
				93.02	10.42	83.48	23.67	84.64	20.30	85.54	21.85
✓				95.10	12.08	85.28	20.33	88.58	20.11	87.33	20.65
✓	✓			94.21	12.08	88.78	18.17	87.25	20.30	88.99	18.89
✓	✓	✓		95.98	10.42	89.11	16.67	87.90	21.41	90.18	18.52
✓	✓	✓	✓	95.27	10.00	91.24	15.00	91.18	14.71	91.16	15.56

**Table 9:** Impact of weight settings on model performance (%).

$\omega_{1}$	$\omega_{2}$	$\omega_{3}$	OCI_Mk(M)		OCM_Mk(I)		OIM_Mk(C)		ICM_Mk(O)	
			**AUC**	**HTER**	**AUC**	**HTER**	**AUC**	**HTER**	**AUC**	**HTER**
0.5	0.5	1	90.12	13.22	84.58	21.62	86.69	18.30	88.74	19.15
1	0.5	0.5	93.20	14.18	88.18	20.88	89.24	16.11	90.13	17.65
0.5	1	0.5	95.48	11.18	90.78	16.17	90.25	15.44	90.99	16.32
0.5	0.5	0.5	94.91	11.42	89.45	17.67	88.90	18.41	91.78	16.52
1	1	1	95.27	10.00	91.24	15.00	91.18	14.71	91.16	15.56

## Data Availability

Data are available upon request to the corresponding author.
